# Outcomes of a Randomized Controlled Trial on the Effectiveness of Depression and Anxiety Prevention for Adolescents with a High Familial Risk

**DOI:** 10.3390/ijerph15071457

**Published:** 2018-07-10

**Authors:** Sanne P. A. Rasing, Daan H. M. Creemers, Ad A. Vermulst, Jan M. A. M. Janssens, Rutger C. M. E. Engels, Ron H. J. Scholte

**Affiliations:** 1 Child & Adolescent Psychiatry, GGZ Oost Brabant, P.O. Box 3, 5427 ZG Boekel, The Netherlands; d.creemers@ggzoostbrabant.nl; 2 Child and Adolescent Studies, Utrecht University, P.O. Box 80140, 3508 TC Utrecht, The Netherlands; 3 Behavioural Science Institute, Radboud University, P.O. Box 9104, 6500 HE Nijmegen, The Netherlands; a.vermulst@home.nl (A.A.V.); j.janssens@bsi.ru.nl (J.M.A.M.J.); r.scholte@bsi.ru.nl (R.H.J.S.); 4 Erasmus School of Social and Behavioural Sciences, Erasmus University Rotterdam, P.O. Box 1738, 3000 DR Rotterdam, The Netherlands; rutger.engels@cvb.eur.nl; 5 Praktikon, P.O. Box 6909, 6503 GK Nijmegen, The Netherlands

**Keywords:** depression, anxiety, adolescents, prevention, parental psychopathology, randomized controlled trial

## Abstract

A randomized controlled trail was conducted to examine the effectiveness of a depression and anxiety prevention program ‘Een Sprong Vooruit’ (A Leap Forward) among adolescent girls with a high familial risk (*N* = 142). The results showed neither effects of the prevention program directly after the intervention, nor at 6 or 12 months follow-up on depression and anxiety symptoms. Further, latent growth curve modeling (LGCM) was used to examine whether the growth functions for the intervention and the control condition were different. The slope representing the change in depression symptoms was not significantly different between the intervention and the control condition. For anxiety symptoms, the difference between slopes was also not significant. Based on these results, we suggested that these high-risk adolescent girls might benefit more from a more intensive prevention program.

## 1. Introduction

Depression and anxiety are common mental health disorders and are among the most costly health problems [1,2,3]. During adolescence, the rates of depression and anxiety rise dramatically [4]. Among 13 to 17-year old females, the lifetime prevalence of depression is estimated on 16.8% [5]. For anxiety disorders, lifetime prevalence in the same age group is estimated on 38.3% [5]. These numbers only account for clinical diagnoses of these disorders, and adolescents with elevated symptoms below the clinical thresholds are not considered.

Children of parents with a depressive disorder are three times more likely to develop depression symptoms compared to children of parents without mental health problems [6,7]. For children of parents with an anxiety disorder, it is up to seven times more likely that they develop anxiety symptoms [7,8]. Besides the parental psychopathology itself, dysfunctional parenting behavior is found to be associated with an increased risk for depressive and anxiety disorders in offspring [9,10]. Especially negative and affectionless behavior in parents is related to emotional problems in children [11]. Because of the risk of parental psychopathology on emotional problems, it is important to aim prevention programs on youth with parents with mental health problems.

The presence of depression or anxiety disorders at a young age is strongly associated with psychopathology later in life [12,13,14,15]. Suffering from emotional problems during adolescence, but also having elevated symptoms, is negatively related to social and family functioning [16,17], associated with poor academic performance [15,17,18], related to depression and anxiety in later life [4,19], and can even lead to suicide [20,21].

Because of detrimental consequences of developing depression and anxiety during adolescence, reducing the risk and decreasing potential elevated symptoms of depression and anxiety is pivotal. Adolescence seems, therefore, an important life phase to implement depression and anxiety prevention programs. Recent review studies found that there is less evidence for the effectiveness of universally delivered depression prevention in adolescence [22,23]. Further, the effects of universally delivered anxiety prevention are notoriously low, and in particular lower than the effects of selective and indicated prevention [23,24,25]. Prevention programs that are selective or indicated, and thus focus on adolescents at risk for developing depression or anxiety, have shown to reduce depression or anxiety symptoms or reduce the incidence of depression or anxiety disorders. The reason might be that high-risk individuals, participating in selective or indicated prevention programs, are more effectively engaged in the prevention program because they are motivated by their distress, which is not the case in non-risk individuals [26].

Depression and anxiety disorders in adolescents are known to have a large comorbidity, shown in high diagnostic comorbidity rates [27,28] as well as in highly correlated symptoms [29], and the co-occurrence of depression and anxiety symptoms is related to greater severity in symptoms [30]. Further, research has shown that depression and anxiety are characterized by similar negative cognitions, negative affect, and elevated distress [31,32]. By focusing on transdiagnostic underlying mechanisms of both depression and anxiety, and therefore providing intervention strategies for symptoms of both disorders, there is potentially a larger benefit of prevention programs [31,33]. Cognitive behavioral therapy has shown to be a highly efficacious treatment of depression and anxiety [34]. The techniques used in cognitive behavioral therapy are known to target the underlying mechanisms of both depression and anxiety, such as to improve negative affect, decrease distress, and increase of cognitive coping by experiencing more positive cognitions [35]. Prevention programs departing from cognitive behavioral therapy might therefore be appropriate for adolescents to decrease elevated depression and anxiety symptoms and to prevent the onset of both depression and anxiety disorders.

The present study evaluated the effects of a prevention program aimed at the prevention of depression as primary outcome and anxiety as secondary outcome for adolescent girls with a high familial risk in a randomized controlled trial. Females are known to have a heightened risk on developing depression or anxiety symptoms [5]. Differences between males and females arise during adolescence and females show more often depression and anxiety symptoms [5] and are more vulnerable to develop depression and anxiety disorders [36]. To examine whether the program was effective, we tested effects directly after the preventive intervention and long-term effects after six and twelve months for depression symptoms and anxiety symptoms separately. The main hypothesis was that participants in the intervention condition would show a lower level of depression and anxiety symptoms compared to participants in the control condition.

## 2. Method

### 2.1. Ethics

The study was approved by the medical ethics committee CMO Region Arnhem-Nijmegen, The Netherlands (NL41344.091.12). All participants provided written informed consent. The trial was registered in the Dutch Trial Register (NTR) as NTR3720. Results of this study were reported in accordance with the CONSORT 2010 statement for reporting parallel group randomized trials [37].

### 2.2. Procedure

A preventive intervention aimed at preventing depression and anxiety in adolescent girls with high familial risk was examined with a non-blinded randomized controlled trial with two conditions (intervention versus control) [38]. A total of 862 female adolescents in the first and second year of five secondary schools located in rural regions in the middle and south of the Netherlands, from vocational level up to pre-university level, gave passive consent and were screened on depression symptoms using the Children’s Depression Inventory 2 (CDI-2) [39], anxiety symptoms using the Spence Children’s Anxiety Scale (SCAS) [40], suicidal ideation using one item from the CDI-2, and perceived parental psychopathology according to the adolescents using a self-developed instrument. One-hundred-and-seven female adolescents declined screening. Inclusion criteria were being aged between 11–15 years old, having increased levels of depression (CDI-2 ≥ 15) and/or anxiety (SCAS ≥ 39), having at least one of the parents with parental psychopathology based on adolescents’ perception, and having sufficient knowledge of the Dutch language. Exclusion criteria were the absence of parental permission, adolescent already receiving treatment for mental health problems, and presence of prominent suicidal ideation (score 2 on CDI-2 item: a desire to kill oneself, if given the chance).

Of these 862 girls, 163 met the eligibility criteria of which three could not be reached, one received no parental permission, six already received care, and eleven declined participation. In total, 142 girls gave written informed consent together with their parents; 69 participated in the intervention condition and 73 in the control condition. Participants in the intervention condition were assessed at baseline (T0), after two sessions (T1), after four sessions (T2), post-intervention (T3), at 6 months (T4) and 12 months (T5) follow-up. Participants in the control condition were assessed at the same time points. In addition, they were offered to follow the preventive intervention as soon as the study ended. Participants in both conditions received rewards when they completed all assessments.

### 2.3. Sample Size

A power calculation indicated that to detect a medium effect size (Cohen’s *d* = 0.50) on depression symptoms at 12 months follow-up using a two-tailed test with α = 0.05 and power (1−β) = 0.80, 64 participants were needed per condition. We accounted for potential attrition and missing data, and therefore, we increased the sample size by 25%, resulting in 160 participants (80 in the intervention condition and 80 in the control condition).

### 2.4. Participants

One-hundred-and-forty-two female adolescents with elevated depression or anxiety symptoms participated in this effectiveness trial. The adolescents were aged 11–14 years (*M* = 12.87, *SD* = 0.69), and were in the first or second year of secondary education. Educational levels were: vocational training (18.3%), vocational training/high school training (17.6%), high school training (15.5%), high school training/pre-university training (30.3%), and pre-university training (18.3%). Most adolescents had the Dutch nationality (97.2%), and the remaining adolescents (2.8%) had a different European and non-European origin. Other baseline characteristics of the participants are shown in Table 1.

### 2.5. Randomization

Directly after screening, participants were randomly allocated to the conditions, stratified on school, grade and educational level (allocation ratio (1:1)). The randomization was done by an independent researcher using a computer-generated randomization procedure.

### 2.6. Intervention

The prevention program ‘Een Sprong Vooruit’ (A Leap Forward) was developed for adolescents with parents with mental health problems and focuses on depression and anxiety prevention. The program consists of six sessions, each 90 min and it mainly uses techniques based on cognitive behavioral therapy, behavioral activation, and exposure, of which we know are effective in treating depressive and anxiety disorders. The length of the program is a balance between a short program with only few sessions to keep drop-out as low as possible and a long program with more sessions to maximize usage of elements that are known to affect the symptoms. Adolescents will learn about emotions and how to recognize them; relationships between activating events, beliefs and consequences; strategies to replace pessimistic beliefs with optimistic beliefs; influence emotions with behavior; divide fearful activities into small steps and experience the decrease in anxiety; and how to get support from the social environment. The program ‘Een Sprong Vooruit’ uses components of the Dutch depression prevention program ‘Op Volle Kracht’ [41,42,43], which is an adapted version of the Penn Resiliency Program [44], components of the Dutch anxiety treatment protocol ‘Denken + Doen = Durven’ [45], and is inspired by the Friends program [46]. A detailed description of the program can be found in the study protocol [38].

Group size of the intervention groups varied between 6 and 12 adolescents per group (group size *M* = 8.63, *SD* = 1.85), and the participants attended an average of 5.29 (1.34) sessions (median 6; range 0–6). Therapists were at least psychologists at master level. Further, treatment integrity was measured through a self-report questionnaire assessing which parts of the total program were actually delivered, i.e., how many instructions and exercises the program were actually given to and done by the participants. We calculated that therapists delivered an average of 95% of the program (range 91–98%).

### 2.7. Measures

The Children’s Depression Inventory 2 (CDI-2) was used to measure depression symptoms [39]. This questionnaire contained 28 items, each consisting of three statements graded in severity from 0 to 2. Sample statements included “Sometimes I feel sad”, “Most of the times I feel sad”, and “I always feel sad”. Sum scores were computed by adding together scores of all 28 items. Cronbach’s alpha was 0.71 at screening, 0.81 on T0, 0.83 at T1, 0.90 at T2, 0.89 at T3, 0.91 at T4, and 0.92 at T5. One of the items of this questionnaire was used to assess suicidal ideation (Kovacs, 2012). The item has score of 0 (“no suicidal ideation”), 1 (“thoughts of wanting to kill oneself, with no intent to do so”), and 2 (“a desire to kill oneself, if given the chance”). Participants with a score of 2 were directly referred to a therapist within an institute of mental health care.

The Spence Children’s Anxiety Scale (SCAS) was used to measure anxiety symptoms [40]. The 44 items of this self-report questionnaire are rated on a 4-point scale ranging from “never” to “always”. Sample statements were “I worry about things” and “I am scared of the dark”. Sum scores were computed by summing up scores of all items, excluding the filler 11, 17, 26, 31, 38, and 43 (e.g., “I am popular amongst other kids of my own age”). Cronbach’s alpha was 0.81 at screening, 0.87 at T0, 0.91 at T1, 0.90 at T2, 0.92 at T3, 0.92 at T4, and 0.94 at T5.

The self-developed measure perceived parental psychopathology was used during the screening of the female adolescents in order to assess whether parents had a history of or showed mental health problems at that time [47]. Students responded to seven statements about parental psychopathology for both mother and father, for example: “My parent received treatment from a psychologist or psychiatrist”, “My parent had a decreased interest or pleasure in most or all activities”, with wording of parent was replaced by either mother or father. Answers were rated as not present (0) or present (1). We counted presence of one item as presence of perceived parental psychopathology. This measure will be referred to as perceived parental psychopathology.

The Brief Symptoms Inventory (BSI) was used to measure parental psychopathology [48]. The self-report questionnaire is a short version of the Symptom Checklist-90 (SCL-90) [49] and measures general symptoms of psychopathology. The 53 items were rated on a 5-point scale from “not at all” to “extremely”. Sample statements were “Feeling no interest in things” and “Feeling tense or keyed up”. Both mothers and fathers rated the questionnaire, resulting in scores on maternal and paternal psychopathology. Mean scores were computed by averaging all items. Parental psychopathology was measured at T0. Cronbach’s alpha was 0.94 for maternal psychopathology and 0.93 for paternal psychopathology. This measure will be referred to as parental psychopathology.

### 2.8. Statistical Analyses

We conducted independent *t*-tests to analyze differences in the primary outcome measure depression symptoms at all time-points. Further, we did the same for the secondary outcome measure anxiety symptoms.

We applied latent growth curve modeling (LGCM) to examine the changes in the primary outcome measure depression symptoms and the secondary outcome measure anxiety symptoms over time, using Mplus version 6.11 [50]. To estimate the parameters in the models, we applied the maximum likelihood estimator (ML). Using the full information maximum likelihood estimator has shown to be the preferred method for handling missing data [51]. Model fit was assessed by χ^2^, Comparative Fit Index (CFI: preferably 0.95 or higher), and Root Mean Square Error of Approximation (RMSEA: preferably 0.05 or lower, and satisfactory between 0.05 and 0.08) [52].

To examine the changes in depression and anxiety symptoms, we first tested models, separately for depression and anxiety symptoms, without predictors. In these models, the mean of the intercept provides information about the average level of depression or anxiety symptoms and the mean of the slope represents the average change in depression or anxiety symptoms across the time-points. Second, we added condition (i.e., whether the adolescents participated in the intervention or control condition) to the models and tested separately for depression and anxiety symptoms whether the growth functions for the intervention and the control condition were different.

## 3. Results

### 3.1. Descriptive Statistics

Means, standard deviations, and *t*-values for differences on depression symptoms between the intervention and control condition were calculated (Table 2). As can be seen in Table 2, the level of depression symptoms was not significantly different at baseline between participants in the intervention condition compared to participants in the control condition. This is also the case for the levels of depression symptoms during the intervention, after the intervention, and during follow-up. The within-group effect sizes for change in total depression symptoms from baseline to 12-month follow-up showed a small effects size (Cohen’s *d* = 0.35) in the intervention condition and no effect (Cohen’s *d* = 0.18) in the control condition. Between-group difference in depression symptoms at 12-month follow-up showed no effect (Cohen’s *d* = 0.16). Measured at screening, depression symptoms in adolescents were not correlated to perceived parental psychopathology in mothers (*r* = −0.05, *p* = 0.58) and fathers (*r* = 0.04, *p* = 0.67). At baseline, depression symptoms were neither correlated to parental psychopathology in neither mothers (*r* = −0.03, *p* = 0.75) nor fathers (*r* = 0.13, *p* = 0.20). Perceived maternal psychopathology at screening showed to be significantly associated to maternal psychopathology at baseline (*r* = 0.22, *p* = 0.01) and perceived paternal psychopathology at screening was significantly associated to paternal psychopathology at baseline (*r* = 0.39, *p* < 0.001). 

Further, means, standard deviations, and *t*-values for anxiety symptoms were calculated between the intervention and control condition (Table 3). The level of anxiety symptoms was not significantly different between participants in the intervention condition and control condition at baseline, and neither during the intervention, after the intervention, and during the follow-up. The within-group change in anxiety symptoms from baseline to 12-month follow-up showed a medium effect size (Cohen’s *d* = 0.58) in the intervention condition and a medium effect size (Cohen’s *d* = 0.52) in the control condition, implying that both groups decreased substantially in symptom level. Between-group difference in anxiety symptoms at 12-month showed no effect (Cohen’s *d* = 0.05). Measured at screening, anxiety symptoms in adolescents were not correlated to perceived parental psychopathology in mothers (*r* = 0.08, *p* = 0.37) and showed a small positive correlation to perceived parental psychopathology in fathers (*r* = 0.17, *p* = 0.048). At baseline, anxiety symptoms were not significantly correlated to parental psychopathology in neither mothers (*r* = −0.04, *p* = 0.69) nor fathers (*r* = 0.04, *p* = 0.65).

**Figure 1:**
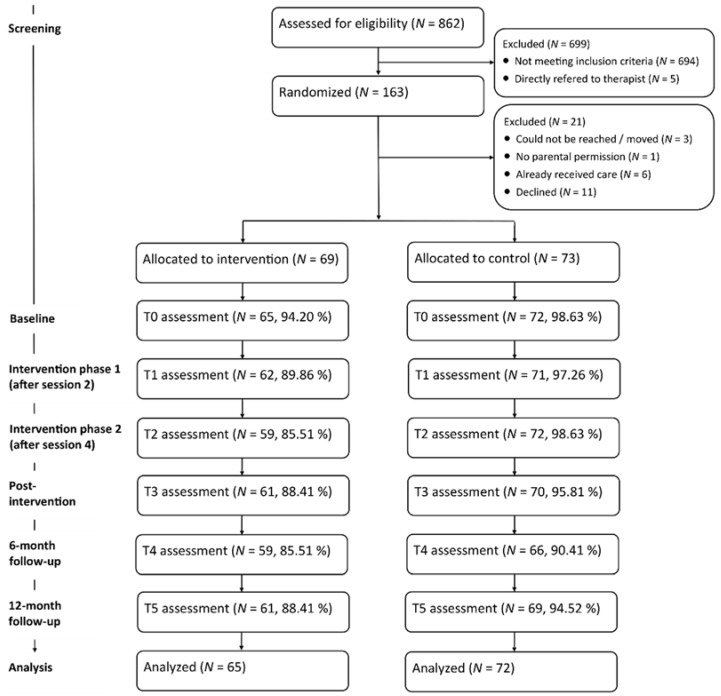
Flow diagram of participants.

The retention rates were high with 137 (96.5%) adolescents completing the baseline assessment (T0), 133 (93.7%) completing the intervention phase 1 assessment (T1), 131 (92.3%) completing the intervention phase 2 assessment (T2), 131 (92.3%) completing the post-intervention assessment (T3), 125 (88.0%) completing the 6-month follow-up assessment (T4), and 130 (91.5%) completing the 12-month follow-up assessment (T5). Figure 1 presents the flow diagram of participants.

### 3.2. Model Findings

First, the linear growth model for depression symptoms was tested. All six measures of depression symptoms from baseline to 12-month follow-up were used to estimate a growth curve. The model showed an acceptable fit (χ^2^ (15, *N* = 138) = 36.16, CFI = 0.97, and RMSEA = 0.10). Adding a quadratic slope did not improve the model fit, and therefore we decided to use the linear model. It is known that for small samples cut-off points for RMSEA are too restrictive [53] and acceptable models might be over rejected [54]. Unfortunately, Mplus does not provide individual fit indices. Therefore, the model was accepted. Next, we added condition as grouping variable to the model (χ^2^ (32, *N* = 138) = 66.25, CFI = 0.95, and RMSEA = 0.13). The intercept (*B* = 14.26, *p* < 0.001) and slope (*B* = −0.21, *p* < 0.001) of the intervention condition were both significant, showing that the depression symptoms were significantly different from zero at baseline and decreased significantly over time. The intercept (*B* = 14.41, *p* < 0.001) of the control condition was significant, but the slope (*B* = −0.11, *p* = 0.09) showed not to be significant, indicating that the depression symptoms were significantly different from zero at baseline and did not decrease over time. Finally, a Wald χ^2^ test (χ^2^ (1, *N* = 138) = 0.02, *p* = 0.89) showed that the intercepts of depression symptoms were not significantly different between the intervention and control condition. A Wald χ^2^ test for the slopes (χ^2^ (1, *N* = 138) = 1.46, *p* = 0.23) showed that the slopes of depression symptoms in the intervention condition and the control condition were not significantly different.

Second, the model for anxiety symptoms was tested. Again, all six measures of anxiety symptoms from baseline to 12-month follow-up were used in the estimation of the growth curve. The model showed an acceptable fit (χ^2^ (16, *N* = 138) = 68.17, CFI = 0.92, and RMSEA = 0.15). Here again, a quadratic slope did not improve the model fit. As mentioned before, RMSEA is known to be too restrictive in small samples, and, therefore, the model was accepted. In the next step, we added condition as a grouping variable to the model (χ^2^ (10, *N* = 138) = 85.34, CFI = 0.92, and RMSEA = 0.16). The intercept (*B* = 37.00, *p* < 0.001) and slope (*B* = −0.54, *p* < 0.001) in the intervention condition were both significant, indicating that anxiety symptoms were significantly different from zero at baseline and decreased significantly over time. The intercept (*B* = 35.95, *p* < 0.001) and slope (*B* = −0.53, *p* < 0.001) in the control condition were also both significant, showing that the anxiety symptoms at baseline were significantly different from zero and that they significantly decreased over time. Finally, a Wald χ^2^ test (χ^2^ (1, *N* = 138) = 0.20, *p* = 0.66) for intercepts showed no significant difference between the intercepts of anxiety symptoms in the intervention condition and control condition. A Wald χ^2^ test (χ^2^ (1, *N* = 138) = 0.002, *p* = 0.96) showed that the slopes of anxiety symptoms were not significantly different between the intervention and control condition.

## 4. Discussion

In the present study, a randomized controlled trial was conducted to examine whether a novel depression and anxiety prevention program ‘Een Sprong Vooruit’ had an effect on depression as primary outcome and anxiety symptoms as secondary outcome in adolescent girls with a high familial risk. In contrast to our hypothesis, participants in the intervention condition did not show a more favorable trajectory on continuous measures of depression and anxiety symptoms than adolescents in the control condition. The symptoms of depression decreased in participants in the intervention condition and symptoms of anxiety decreased in participants in both conditions, although we found no differences between conditions. This means that results showed that the program did not seem to be effective in reducing symptoms. Strikingly, anxiety symptoms decreased significantly in the control condition, without offering any form of intervention.

Comparing the results of the current study to other studies on the effectiveness of indicated prevention gives us some interesting insights. Unlike findings in other studies on depression and anxiety in adolescents with elevated symptoms [43,55], and a meta-analysis on the effectiveness of depression prevention in children with parents with a depressive disorder [56], we found no greater decrease in depression and anxiety symptoms in the intervention condition than in the control condition. Our program was based on evidence-based treatment techniques for depression [57,58] as well as for anxiety disorders [59], and, therefore, we expected to find effects on both depression and anxiety symptoms. There are several possible reasons for the lack of effect of the intervention in the present study.

First, it can be the case that the program lacks focus on one technique, as it covers several techniques in a relatively short time (six sessions of 90 min). That is, in the program adolescents were trained in psycho-education, cognitive restructuring, behavioral activation, exposure, and strengthening their social network. It could be that six sessions provide too little opportunity to become well trained and therefore these techniques and the program as such, failed to reduce symptom levels. Research suggests that the ability to incorporate techniques and processes offered by the interventions is depending on the number of sessions of the intervention, and that eight sessions of one hour (i.e., 480 min) might be insufficient [60]. Our program consists of six sessions of 90 min and the total duration (540 min) is longer than the advised minimum. However, the fact that the program contains several techniques means that the amount of time per technique may not be sufficient to influence the development of depression or anxiety symptoms. Especially in the larger groups (i.e., 12 adolescents per group), there was little time to practice and to individually tailor the examples during the intervention.

A second possible reason for a lack of intervention effects is the quality of the match between the participants and the prevention program. The program was developed as an efficient and light program for both depression and anxiety prevention. Therefore, the program contains elements for both depression and anxiety, as we mentioned before. Adolescents were included for their risk on depression and anxiety based on elevated depression and/or anxiety symptoms. However, only slightly more than 40% of the adolescents suffered from both elevated depression and anxiety symptoms. This means that almost 60% showed only depression or anxiety symptoms, and were during the program confronted with exercises that did not match with their needs, and these adolescents might have profited more from a program specifically focused on either of them. This allows participants to practice more with skills aimed at their own problems and prevention might be more effective for these adolescents.

A third reason for a lack of effect might be the significant decrease in symptoms in adolescents in the control condition. There are a multiple explanations for this decrease. Firstly, it might be explained by the number of assessments the adolescents received. Adolescents in the intervention condition received two assessments during and three assessments after the intervention and adolescents in the control condition received assessments with the same interval. This might have led to awareness and contributed to the decrease of symptoms in the control condition. A similar decrease in symptoms in the control condition are described in several other studies [61,62]. Also, the adolescents received attention of the researchers through telephone and email and this may have had a therapeutic effect [63]. Secondly, the awareness of being observed and evaluated is known for its decreasing effect on symptoms and is called the Hawthorne effect [64,65]. When participants know they are being studied, they change their behavior and in this study this may have resulted in a decrease in symptoms.

Strengths of this study were the long-term follow-up measurements and the high retention rates. Further, the prevention program was strongly based on theories about treatment techniques for depression and anxiety. In addition, the group sessions of the prevention program showed high treatment integrity. Therefore, we are allowed to interpret our results about effectiveness with confidence [66]. Future should consider using independent ratings of treatment fidelity, for example rating video-recordings of the sessions by an independent therapist. However, some limitations should be mentioned. This study intended to measure changes in depression and anxiety symptoms during the intervention. We assessed depression and anxiety symptoms two times during the intervention, namely following the second session and following the fourth session. Yet, reporting too frequently on their own depression and anxiety symptoms might have led to awareness of their mental wellbeing and that in turn might have led to a reducing effect on symptoms. This may have led to an unintended and unforeseen effect in the control condition. Further, data were collected only through self-report questionnaires, often mentioned to be subjective in measuring symptoms. Future studies should consider adding a more objective measure of depression and anxiety, for example using a clinical interview. Another suggestion is to include other factors that could identify risk, such as emotional support of parents or conflict within the family.

## 5. Conclusions

In conclusion, the present study showed no intervention effects of ‘Een Sprong Vooruit’ in reducing depression or anxiety symptoms compared to the control condition. We argued that prevention programs should have a clear focus on either depression or anxiety prevention, resulting in more time to practice skills to target these problems. Further, we recommended to target adolescents with less heterogeneity in their symptoms (i.e., either depression or anxiety), so that they could benefit from the complete program. Taking these suggestions into account, prevention program might be more effective in improving mental health of adolescents.

## Figures and Tables

**Table 1:** Baseline characteristics.

Variable	Total Sample (*N* = 142)		Intervention Condition (*N* = 69)		Control Condition (*N* = 73)		*t*	*p*
	*M*	*SD*	*M*	*SD*	*M*	*SD*		
*Adolescents*								
Age	12.87	0.69	12.84	0.73	12.90	0.66	−0.51	0.61
Nationality								
Dutch	97.2%		97.1%		97.3%			
Other	2.8%		2.9%		2.7%			
Educational level								
Vocational training	18.3%		20.3%		16.5%			
Vocational/high school training	17.6%		17.4%		17.8%			
High school training	15.5%		17.4%		13.7%			
High school/pre-university training	30.3%		30.4%		30.1%			
Pre-university training	18.3%		14.5%		21.9%			
Symptom level								
Depression symptoms	16.08	5.46	15.93	4.91	16.23	5.97	−0.33	0.74
Anxiety symptoms	45.76	12.24	47.78	12.14	43.85	12.12	1.93	0.06
*Mothers*								
Age	43.89	4.41	44.03	4.19	43.76	4.63	0.32	0.75
Perceived maternal psychopathology	1.89	1.39	1.97	1.40	1.82	1.38	0.64	0.52
Maternal psychopathology	0.33	0.30	0.33	0.30	0.32	0.30	0.16	0.89
*Fathers*								
Age	47.10	4.58	47.40	4.74	46.80	4.44	0.63	0.53
Perceived paternal psychopathology	1.11	1.21	0.99	1.01	1.22	1.37	−1.17	0.24
Paternal psychopathology	0.25	0.25	0.26	0.24	0.24	0.26	0.32	0.75

**Table 2:** Means, standard deviations, and *t*-values for differences on depression symptoms between the intervention and control condition.

Variable	Total Sample (*N* = 142)		Intervention Condition (*N* = 69)		Control Condition (*N* = 73)		*t*	*p*
	*M*	*SD*	*M*	*SD*	*M*	*SD*		
Depression symptoms screening	16.08	5.46	15.93	4.91	16.23	5.97	−0.33	0.74
Depression symptoms T0	14.44	6.50	14.32	5.89	14.54	7.04	−0.20	0.85
Depression symptoms T1	14.49	6.92	14.60	6.53	14.39	7.29	−0.17	0.87
Depression symptoms T2	14.07	8.40	13.80	6.92	14.29	9.48	−0.35	0.73
Depression symptoms T3	13.66	8.06	13.36	7.65	13.91	8.45	−0.39	0.70
Depression symptoms T4	13.04	8.96	11.98	7.84	13.98	9.82	−1.25	0.21
Depression symptoms T5	12.38	9.12	11.62	9.03	13.06	9.21	−0.90	0.37

**Table 3:** Means, standard deviations, and *t*-values for differences on anxiety symptoms between the intervention and control condition.

Variable	Total Sample (*N* = 142)		Intervention Condition (*N* = 69)		Control Condition (*N* = 73)		*t*	*p*
	*M*	*SD*	*M*	*SD*	*M*	*SD*		
Anxiety symptoms screening	45.76	12.24	47.78	12.14	43.85	12.12	1.93	0.06
Anxiety symptoms T0	37.77	13.58	38.97	13.51	36.69	13.62	0.98	0.33
Anxiety symptoms T1	37.29	15.78	37.52	16.52	37.10	15.23	0.15	0.88
Anxiety symptoms T2	35.69	15.11	36.57	15.37	34.99	14.98	0.59	0.55
Anxiety symptoms T3	33.19	16.03	33.92	16.41	32.57	15.79	0.48	0.64
Anxiety symptoms T4	32.81	16.18	32.72	16.19	32.89	16.23	−0.06	0.95
Anxiety symptoms T5	29.39	16.81	29.83	17.71	29.00	16.10	0.28	0.78
